# 3C suppresses PINK1-mediated mitophagy and contributes to coxsackievirus B3 replication

**DOI:** 10.1080/21505594.2026.2662767

**Published:** 2026-04-26

**Authors:** Tingjun Liu, Ao Wan, Yinhai Xu, Hongxiang Lu, Yiwei Xie, Han Wu, Jiang Wang, Hua Wang, Tingting Hao, Yonggen Zhang, Jinfeng Xu, Hongxing Shen, Shibao Li

**Affiliations:** a Department of Laboratory Medicine, The Affiliated Hospital of Xuzhou Medical University, Xuzhou, China; b School of Medical Technology, Xuzhou Medical University, Xuzhou, China; c Department of Laboratory Medicine, The people’s Hospital of Jiawang, Xuzhou, China; d Department of Laboratory Medicine, Jiangning Hospital Affiliated to Nanjng Medical University, Nanjing, China; e Department of Laboratory Medicine, School of Medicine, Jiangsu University, Zhenjiang, China; f Department of Clinical Laboratory, Zhenjiang Center for Disease Control and Prevention, Zhenjiang, Jiangsu, China

**Keywords:** Viral myocarditis, coxsackievirus B3, PINK1, MAVS, mitophagy

## Abstract

Viral myocarditis (VM) is a cardiac inflammatory condition caused by viral infection and serves as a critical precursor to life-threatening complications, such as dilated cardiomyopathy and heart failure. Coxsackievirus B3 (CVB3), a predominant etiological agent of VM, lacks targeted therapeutic interventions despite ongoing antiviral development. Mitophagy is a selective mitochondrial quality control mechanism mediated by PINK1. It has two key roles: maintaining mitochondrial homeostasis and regulating innate antiviral immunity. Here, we employed single-cell RNA sequencing to reveal a significant correlation between impaired mitophagy and cardiomyocyte pathology in CVB3-induced myocarditis. We demonstrated that CVB3 infection suppresses PINK1-dependent mitophagy, while the attenuation of PINK1 reciprocally enhances CVB3 replication. Mechanistically, CVB3 non-structural protein 3C promotes the degradation of mitochondrial antiviral signaling protein (MAVS). MAVS interacts with PINK1 to form a regulatory loop: PINK1 deficiency boosts MAVS reduction, which further promotes viral replication and worsens myocardial injury. Furthermore, we identify the transcription factor FOSL1 as a novel negative regulator of PINK1 transcription through direct promoter binding. Collectively, these findings show that the 3C/FOSL1/PINK1/MAVS signaling axis is a key mechanism in CVB3 pathogenesis. We propose innovative therapeutic targets for viral myocarditis through restoration of mitochondrial homeostasis and modulation of host-virus interactions.

## Introduction

Myocarditis, an inflammatory disorder of myocardium, poses significant clinical challenges due to its heterogeneous progression. While a majority of patients achieve spontaneous recovery, approximately 20% develop chronic sequelae, including dilated cardiomyopathy (DCM) and congestive heart failure, underscoring the need to elucidate pathogenic mechanisms driving disease progression [1]. Viral infections, particularly those caused by Coxsackievirus B3 (CVB3), represent the predominant etiology of viral myocarditis (VM). As a member of the *Picornaviridae* family, CVB3 is a non-enveloped, single-stranded positive-sense RNA virus whose replication cycle critically depends on the proteolytic activity of viral protease 3C. This enzyme processes the viral polyprotein into mature structural and non-structural components essential for replication, assembly, and host cell egress [2].

Autophagy refers to the catabolic process where cellular components, including cytosol, organelles and protein aggregates, are encapsulated by a double-membraned structure called the autophagosome. It can be categorized into two types: nonselective autophagy and selective autophagy. Nonselective autophagy is induced upon nutrient deprivation, providing cells with essential metabolic building blocks and energy [3,4]. By contrast, mitophagy (mitochondrial selective degradation), a selective autophagy pathway responsible for mitochondrial quality control, maintains cellular homeostasis by eliminating depolarized mitochondria and recycling metabolic substrates such as nucleotides, amino acids, and lipids [5]. In selective autophagy, the specificity is guaranteed by stress-dependent specific labeling of individual cargoes and the involvement of receptors [6].

Receptors are defined as a class of proteins that bind both the cargo and autophagy-related (ATG) proteins and are classified into two types: ubiquitin-binding receptors, which recognize the ubiquitin chains of cargo, and cargo-localizing receptors, which directly localize to the cargo to be degraded. Among the known ubiquitination events in depolarized mitochondria, the ubiquitination of outer mitochondrial membrane proteins mediated by the E3 ubiquitin ligase Parkin and PTEN-induced kinase 1 (PINK1) is the most well-characterized [7,8]. Parkin is widely expressed in various tissues, including the brain, skeletal muscle, heart, and liver, indicating its extensive physiological function [9]. Upon cellular stimulation, Parkin transfers from the cytoplasm to mitochondria, and this recruitment process is mediated by PINK1. Subsequent ubiquitination of mitochondrial substrates marks damaged organelles for autophagic clearance [5,10]. Studies have shown that mitophagy plays a regulatory role in infections caused by various viruses, such as influenza A virus (IAV), Zika virus, and others [11,12]. CVB3 infection promotes mitochondrial fission by disrupting the mitochondrial network. As shown by mitochondrial quantification, the mitochondrial area was reduced by 4-fold in CVB3-infected cells [13]. CVB3 blocked autophagic flux by inhibiting autophagosome-lysosome fusion.

In CVB3-induced VM, CVB3, which blocked autophagic flux by inhibiting autophagosome-lysosome fusion, promotes cardiomyocyte mitophagy and suppresses inflammasome activation ,attenuating macrophage infiltration-induced cardiomyocyte injury in VM [14]. Promoting mitochondrial membrane depolarization and fission could enhance CVB3 release by extracellular vesicles. PINK1 knockout mice exhibit exacerbated viral replication, myocardial injury, and mortality compared to wild-type counterparts, suggesting a protective role for mitophagy in CVB3 infection [15]. In other research, silencing PINK1 suppressed CVB3 RNA expression and TCID_50_. Notably, Parkin silencing reduced mitophagy, aggravated mortality and accelerated the development of cardiac dysfunction in CVB3-treated mice [16]. Paradoxically, CVB3 suppresses PINK1 and Parkin expression, attenuating mitophagy-a phenomenon whose molecular underpinnings remain unresolved [17]. In this study, CVB3 was found to decrease the expression of PINK1 and Parkin, thereby down-regulating mitophagy, and mitophagy suppressed CVB3 replication. But, the underlying mechanisms remain poorly understood.

To address this knowledge gap, we employed single-cell sequencing (scRNA-seq) and identified the PINK1- mediated mitophagy pathway as a key regulatory node in CVB3-infected cardiomyocytes. Building on prior reports that viral pathogens modulate mitophagy to enhance replication and evade immunity [18], we investigated the interplay between CVB3 and PINK1 signaling. Specifically, our research objectives are three-fold: (1) Verify whether CVB3 regulates PINK1-mediated mitophagy to affect virus replication; (2) Elucidate the upstream molecular mediators through which CVB3 modulates PINK1 expression; (3) Confirm the pathological significance of PINK1-mediated mitophagy in CVB3-induced VM, providing a theoretical basis for targeted therapies.

Our findings reveal that CVB3 employs its non-structural protein 3C to transcriptionally downregulate PINK1 via the transcription factor FOSL1, thereby impairing mitophagy and amplifying viral propagation. These results not only delineate a novel mechanism by which CVB3 subverts mitochondrial quality control but also highlight mitophagy as a potential therapeutic target in viral myocarditis.

The central hypothesis of this study is that CVB3 subverts mitophagy to amplify viral propagation by utilizing its non-structural protein 3C to transcriptionally downregulate PINK1 via the transcription factor FOSL1. This work not only delineates a novel mechanism by which CVB3 disrupts mitochondrial quality control but also highlights PINK1-mediated mitophagy as a potential therapeutic target in VM.

## Material and method

### Animal models and experimental design

Specific pathogen-free (SPF) male Balb/c mice (4 weeks, 18–22 g), were obtained from the Experimental Animal Center of Jiangsu University (Zhenjiang, China) and housed under controlled conditions (22 ± 1°C, 12-hour light/dark cycle) with ad libitum access to food and water. Mice were randomly allocated into two groups: Sham (*n* = 5) and VM (*n* = 5). For VM induction, mice received an intraperitoneal injection of CVB3 (10^5^ PFU/mouse) diluted in PBS. Sham controls received PBS alone. The mice were euthanized 7 days after infection, and heart tissue and blood were collected and stored for future use. Mice were anaesthetized with 2% isoflurane (5 min induction, 2% maintenance) and euthanized by cervical dislocation at study endpoints.

### Virus preparation and validation

The CVB3/Nancy [19] strain, used in the study, was a gift from Dr. Ruizhen Chen (Department of Cardiology, Zhongshan Hospital, Shanghai, China). Other viruses used in the study, including EV-71, CV-A6, and CV-A10, were provided by the Zhenjiang Center For Disease Control and Prevention. Cells were infected with viruse, and used Sham cells (without virus) as a negative control. Additionally, GFP-CVB3, a recombinant CVB3 strain expressing green fluorescence protein (GFP), was constructed previously based on the plasmid of pMKS1, which contains the full-length CVB3 genomic complementary DNA. Used pMKS1 vector as a negative control. The pMKS1 plasmid was a gift from Prof. Zhaohua Zhong (Harbin Medical University, Harbin, China) [20,21].

### Cell culture and maintenance

HeLa and HEK-293T cells were kindly provided by Prof. Huaiqi Jing from the National Institute for Communicable Disease Control and Prevention, Chinese Center for Disease Control and Prevention (Beijing, China). AC16 cells were maintained in the cell bank of Xuzhou Medical University. All cell lines were cultured in Dulbecco’s modified Eagle’s medium (DMEM; Gibco, 12,430,104, USA) supplemented with 10% fetal bovine serum (Gibco, 10099141C, USA) and 100 μg/mL penicillin-streptomycin (Gibco, 10,378,016, USA). Cell cultures were maintained at 37°C in a humidified incubator with 5 %CO_2_.

### ScRNA-seq

Mice were randomly divided into two groups: the Control group (Sham, without viral infection) and the experimental group (VM, Viral Myocarditis group, infected with CVB3). At 7 days post CVB3 infection, hearts were harvested from five mice in each group, and the five hearts per group were pooled into a single sample for subsequent analysis. ScRNA-seq was performed on isolated and sorted Balb/c mouse heart cells. Single-cell suspensions were processed using the 10× Genomics Chromium System (10× Genomics, Pleasanton, CA) to construct 3’ gene expression v3.1 libraries, followed by sequencing on an Illumina Noveseq6000 sequencer. Briefly, the 10x Genomics platform employs microfluidics to encapsulate cells and barcoded beads in droplets, forming Single Cell GEMs. Cells within droplets are lysed, allowing mRNA to bind to the barcoded beads. Reverse transcription is then conducted to generate cDNA libraries, with sample indices used to identify sequence origins.

### RNA sequencing and analysis

Total RNA was isolated with TRIzol reagent (Invitrogen, USA), with integrity verified (RIN > 8.0, Agilent Bioanalyzer). Libraries were prepared with the VAHTS Universal V6 RNA-seq Library Prep Kit (NR604) and sequenced (Illumina NovaSeq 6000, PE150). Differentially expressed genes (DEG_S_) were identified using the DESeq2 package [22]. Functional enrichment (GO, Reactome) and GSEA (v4.3.2, Hallmark gene sets) were performed as described [23].

### Hematoxylin and eosin (HE) staining

The heart tissue samples were fixed in 10% formalin (Sigma-Aldrich), paraffin-embedded, and sectioned at 4 μm sections. HE staining was conducted following established protocols [24,25].

### Transmission electron microscopy (TEM)

Cells infected with CVB3 or transfected with the 3C vector were collected and processed as previous descriptions [25].

### Plasmid and oligonucleotides transfection

Cells were plated at 1.5 × 10^5^ cells/well in a 12-well plate. Lipofectamine 3000 (L3000015; Invitrogen, USA) was combined with plasmid or siRNAs in 250 µL of DMEM and incubated at room temperature for 10 minutes before being added to the cells. The siRNA sequences used were as follows: PINK1: 5’-CCAACAGGCTCACAGAG-AA-3,’ SPI1: 5’- UUGGUAUAGCUCUGAAUCGUA-3,’ EGR2: 5’-GGCAUGAUCAACAUUGACATT-3,’ FOSL1: 5’- GCCGCCCUGUACCUUGUAUTT-3.’

### Immunofluorescence (IF) assay

Cells cultured on slides were fixed with 4% paraformaldehyde for at least 30 min, followed by blocking with 5% BSA and permeabilization with 0.5% Triton-X-100 (Sigma-Aldrich, USA). Primary antibodies were incubated overnight at 4 °C, followed by staining with DAPI and fluorescent secondary antibodies (Invitrogen, USA). Fluorescence images were acquired using a Leica fluorescence microscope (Mannheim, Germany).

### Western blot

Western blot was conducted as previous described [24–26]. Briefly, cells were harvested and lysed in RIPA buffer (Invitrogen, USA) containing 1% PMSF. Proteins were resolved on 10% SDS-PAGE and transferred to PVDF membrane (Millipore, USA). Membrane was blocked with 5% nonfat milk and probed with the following primary antibodies: PINK1 (23274–1-AP, Proteintech, China), Parkin (sc-32282, Santa Cruz), TOM20 (11802–1-AP, Proteintech, China), COX4 (11242–1-AP, Proteintech, China), MAVS (81910–1-RR, Proteintech, China), FOSL1 (252421, Abcam, USA), HA (51064–2-AP, Proteintech, China), FLAG (20543–1-AP, Proteintech, China), GAPDH (HRP-60004, Proteintech, China).

### RT–PCR and quantitative PCR

Total RNA was extracted using TRIzol reagent (Invitrogen, USA) according to the manufacturer’s protocol. Reverse transcription was performed using the PrimeScript RT Reagent Kit (Takara, Japan), followed by quantitative Real-Time PCR with TB Green Premix Ex TaqII (Takara, Japan). PCR primers were synthesized by Sangon Biotech (Shanghai, China), with sequences detailed in Table 1.

**Table 1.** The sequence of qPCR primers.

Primer	Sequence (5’-3’)
PINK1	GCCGCAAATGTGCTTCATCT
	AGAGCGTTTCACACTCCAGG
VP1	ATTCAAGGTCCGAGTCAAC
	CTGCTTGTCGTGGTGTTA
3C	GGAGGAAGTGGAGGTTAATG
	TTAGGAAGCCGTATTCTGTG
hsa-SPI1	CCCCTCAGCCATCAGAAGAC
	GCTATGGCTCTCCCCATCAC
hsa-ETV5	CGACACTTGTGTTGTGCCTG
	GGCAATGAAGTGGGCATTGG
hsa-EGR2	ACCGCCTCCTCCTCCTTATT
	GGGTAGGCCAGAGAGGAAGA
hsa-FOSL1	GCCTTGTGAACAGATCAGCC
	CTGCAGGAAGTCGGTCAGTT
Mus-SPI1	GGGTGGACAAGGACAAAGGT
	TCTTCACCTCGCCTGTCTTG
Mus-ETV5	AGCAGCCATGAAGGATTCCC
	CTCGATACATGGTGGGCTCC
Mus-EGR2	TGGACCCAGGTCTCATTCCT
	TAGAGAGTGGCGTGAGTGGA
Mus-FOSL1	CTTGTGCCAAGCATCGACAG
	TACTGGGGATAGGCCAGAGG
hsa-GAPDH	AGGTGAAGGTCGGAGTCAAC
	GGGTGGAATCATATTGGAACA
Mus-GAPDH	TGCCCCCATGTTTGTGATG
	TGTGGTCATGAGCCCTTCC

### Chromatin immunoprecipitation (ChIP) assay and PCR

ChIP assays were conducted with an anti-FOSL1 antibody following the protocol of the ChIP Assay Kit (Cell Signaling Technology). PCR was used to quantify fold enrichment, expressed as a percentage of input chromatin. Primer sequences are listed in Table 2.

**Table 2.** The primers sequences of CHIP-PCR.

Primer	Sequence (5’-3’)
Site 1	GTCTCACTCTGTCACCCAGG
	GCTACTTGGGAGTCTGAGGC
Site 2	GCCCAGGATAGAGTGCAGTG
	AAAAACTAGCTGGGCGTGGT
Site 3	ATGGTGTTTTGTTGGCCAGG
	GTGTGGTAGCTCACAGCCTG
ChIP-GAPDH	TACTAGCGGTTTTACGGGCG
	TCGAACAGGAGGAGCAGAGAGCGA

### Luciferase reporter assay

The PINK1 promoter region was cloned into the PmirGLO vector by Sangon Biotech (Shanghai, China). Luciferase activity was measured using the Dual-Luciferase Reporter Assay System (Promega, WI, USA).

### Reactive oxygen species (ROS) assay

To measure ROS production, cells were incubated with 10 μM 2,’7’-Dichlorodihydrofluorescein (DCFH-DA) (S0033S, Beyotime, China) following the manufacturer’s instructions. Fluorescence images were acquired using a fluorescence microscope. ROS levels were quantified by calculating the percentage of DCFH-DA-positive nuclei.

### Immunohistochemistry (IHC) assay

The IHC assay was performed following the manufacturer’s protocol (Boster Bioengineering, Wuhan, China). Briefly, tissue samples were fixed in 4% paraformaldehyde, embedded in paraffin, and sectioned at 4 μm thickness. Sections were deparaffinized in xylene and rehydrated through a graded ethanol series. Endogenous peroxidase activity was quenched by 3% hydrogen peroxide treatment for 10 minutes. After blocking with 5% BSA, sections were incubated with primary antibody at 4°C overnight. Subsequent steps included incubation with IgG-biotin and SABC, followed by DAB chromogenic development and hematoxylin counterstaining. Stained sections were examined under a light microscope.

### Mitochondrial DNA (mtDNA)

MtDNA was extracted from HeLa and AC16 cells using the Mitochondria Isolation Kit (C3601, Beyotime, China), following the manufacturer’s protocol. The isolated DNA was fragmented into 150–200 bp fragments using ultrasonication. Relative mtDNA levels were quantified via qPCR with primers specified in Supplementary Table S1.

### Statistical and reproducibility

All data are expressed as mean +/-SD. Data were analyzed using SPSS 22.0, and figures were generated using GraphPad Prism 9. Two groups were statistically compared using Student’s *t*-test. Multiple groups were statistically compared using ordinary one-way ANOVA or two-way ANOVA. Differences were considered statistically significant at *p* < 0.05. In the figures, asterisks indicate statistical significance (*P < 0.05, **P < 0.01, ***P < 0.001). All representative images were selected from at least three independent experiments with similar results unless indicated differently in the figure legend.

## Results

### Result 1. A global view of VM scRNA-seq

**Figure 1.**
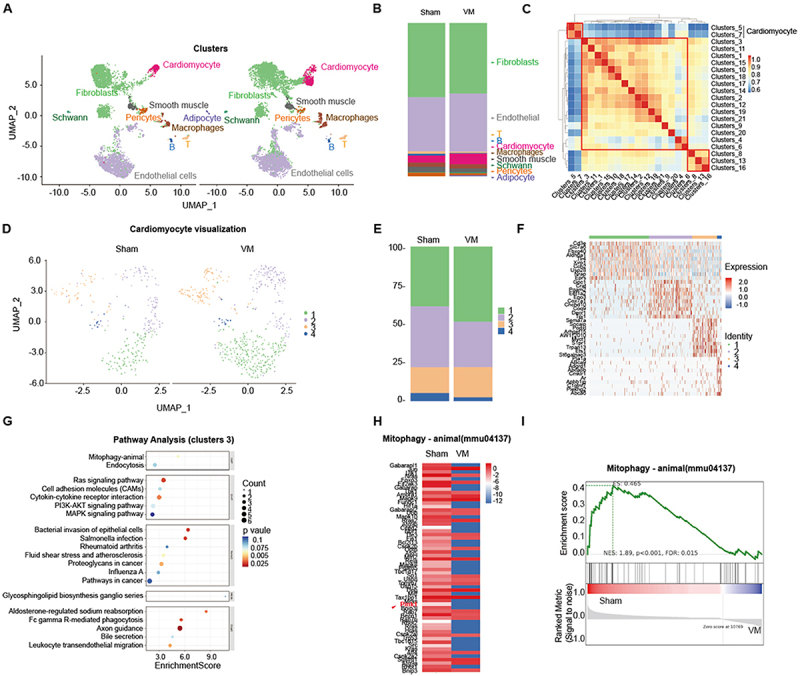
ScRNA sequencing analysis for VM mouse heart tissue.

To delineate cardiac cellular heterogeneity in VM, we conducted scRNA-seq on myocardial tissue from CVB3-infected Balb/c mice (VM group, *n* = 5) and Sham controls (*n* = 5) at 7 days post-infection. Following Cell Ranger quantification and quality control, pooled samples yielded 8,384–11,345 cells per specimen. After stringent filtering of doublets, multiplets, and apoptotic cells, we retained 5,454–7,201 high-quality cells per specimen, with the following metrics: median UMIs/cell (4,691–6,843), genes/cell (1,820–2,390), and mitochondrial UMIs ratio (4.3–7.6%). Unsupervised clustering analysis of 7,201 transcriptome profiles, including both EdU+ nuclei and DAPI singlets, revealed 10 distinct cellular populations through Louvain clustering and UMAP visualization [27] (Figure 1(A)). Based on established markers, these clusters were annotated as fibroblasts, endothelial cells, cardiomyocytes, T cells, B cells, macrophages, smooth muscle cells, Schwann cells, pericytes, and adipocytes (Figure 1(B)).

A UMAP visualization of cardiac cell populations. B Compositional analysis showing proportional distribution of major cell types in sham and VM groups. C Cardiac cell subtype classification based on Spearman correlation analysis. D UMAP projection of re-clustered cardiomyocytes, highlighting four distinct subsets. E Quantitative representation of cell-type proportions within re-clustered cardiomyocytes. F Heatmap displaying the top 10 differentially expressed genes in cardiomyocyte subsets. G KEGG pathway enrichment analysis of cluster-specific genes in Cluster 3 cardiomyocytes. H Single-sample GSEA score heatmap illustrating mitophagy activity across VM samples. I GSEA analysis demonstrating significant suppression of mitophagy pathway enrichment in VM conditions compared to controls.

Chromatin accessibility profiling further segregated cells into three functional clusters (FC) 1–3 based on transcriptomic signatures (Figure 1(C)). FC2 predominated in cardiac tissue, encompassing fibroblasts, endothelial cells, and stromal lineages, while FC1 comprised cardiomyocytes – with sham controls localized to cluster 7 and VM cardiomyocytes enriched in cluster 5. FC3 consisted of immune cells (macrophages, T cells, and B cells) (Figure 1(C)). Sub-clustering of cardiomyocytes identified four subpopulations with distinct group-specific distributions (Figure 1(D)). Clusters 1–4 were all distributed in cardiomyocytes from both the Sham group and the VM group (Figure 1(E)). Cluster-specific top 10 markers are presented in Figure 1(F).

Kyoto Encyclopedia of Genes and Genomes (KEGG) pathway enrichment analysis of Cluster-specific markers showed that each cluster exhibited distinct functional characteristics, with enrichment primarily in pathways related to metabolism, cardiomyopathy, viral infection, and inflammation (Supplementary Figure S1A –S1C). Notably, Cluster 3 was uniquely enriched in the mitophagy pathway (Figure 1(G)). For highly metabolic cells like cardiomyocytes, mitochondrial quality control is particularly indispensable. As a key component of this quality control system, mitophagy thus exerts a crucial role in the onset and progression of myocarditis.

Given the prominence of mitophagy-related pathways in cluster 3, a hallmark of degenerative disorders mediated by PINK1/Parkin, we assessed mitophagy regulation in VM. Gene Set Enrichment Analysis (GSEA) revealed significant suppression of mitophagy-related genes in infected samples (NES = 1.89, nominal *P* < 0.001, FDR = 0.015), with reduced enrichment scores (ES = 0.465) (Figure 1(H,I)). These findings implicate transcriptional repression of mitophagy in CVB3 pathogenesis.

### Result 2. CVB3 downregulation of PINK1 expression to suppressed mitophagy

**Figure 2.**
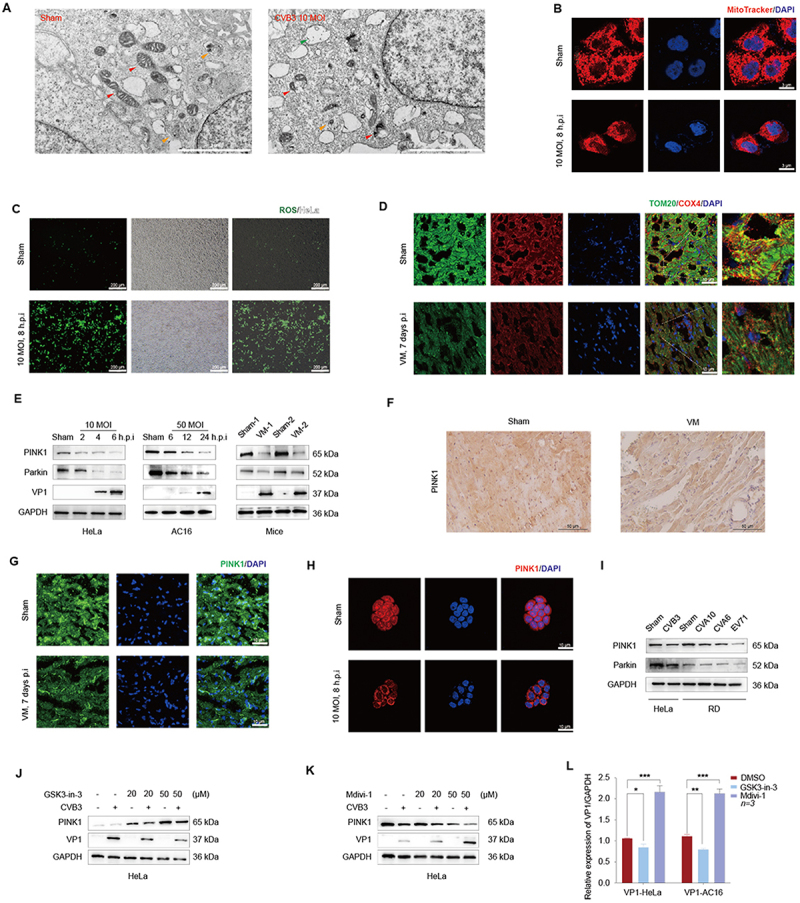
CVB3 **i**nfection disrupts mitochondrial structure and function.

ScRNA-seq highlighted mitophagy pathway enrichment in VM. To validate this, we employed TEM, which revealed mitochondrial abnormalities including membrane invaginations and structural disintegration at 24 h post-infection (p.i) (Figure 2(A)). Mitochondrial mass, assessed via MitoTracker, was significantly reduced in CVB3-infected HeLa cells (Figure 2(B)). Consistent with mitochondria dysfunction, intracellular ROS levels were significantly elevated in infected cells, accompanied by perinuclear clustering, a hallmark of Enteroviral cytopathic effects (Figure 2(C)). This was corroborated by diminished TOM20 and COX4 expression in murine VM models (Figure 2(D)).

A Representative TEM images of CVB3-infected HeLa cells. Red arrows: mitochondria, yellow arrows: lysosomes, and green arrows: CVB3 virus particles. Scale bar = 2 μm. B Mitochondrial fragmentation in CVB3-induced HeLa cells visualized using MitoTracker. Scale bar = 3 μm. C ROS expression in CVB3-infected HeLa cells detected by fluorescence microscopy. Scale bar = 200 μm. D Immunofluorescence staining of TOM20 (green) and COX4 (red) in mouse cardiac tissue, Scale bar = 10 μm. E Western blot analysis of mitophagy-related proteins (PINK1 and Parkin) in CVB3-infected cells and mice. F IHC detection of PINK1 expression in heart tissue of VM mice, Scale bar = 50 μm. G Immunofluorescence staining of PINK1 in mouse cardiac tissue, Scale bars = 10 μm. H Immunofluorescence staining of PINK1 in HeLa cells infected with CVB3 (MOI = 10) for 8 h, Scale bars = 10 μm. I Western blot analysis of mitophagy-related proteins and mitochondrial markers in HeLa cells infected with various Enteroviruses. J Effect of GSK3-in-3 on CVB3 replication assessed by Western blot. K Effect of Mdivi-1 on CVB3 replication assessed by Western blot. L qRT-PCR analysis of VP1 expression levels in CVB3 infected HeLa and AC16 cells. Data were analysed by two-way ANOVA, and the n values represent biological replicates (n = 3/group). Data are presented as mean ±SD. *P < 0.05, **P < 0.01, ***P < 0.001.

Furthermore, the downregulation of PINK1 and Parkin, which are essential proteins for mitophagy, was observed in both *in vitro* and *in vivo* CVB3 infection models (Figure 2(E-H)). Notably, this mitophagy inhibition extended to other enteroviruses (EV71, CA6, CA10; Figure 2(I)), suggesting a conserved mechanism.

GSK3-in-3 is a mitophagy inducer, inducing PINK1/Parkin-dependent mitophagy [28]. After treating HeLa cells, we observed that the expression of PINK1 increases in a dose-dependent manner. Additionally, following infection with CVB3, the expression of PINK1 is downregulated; meanwhile, GSK3-in-3 inhibit the replication of CVB3 (Figure 2(J,L)). Mdivi-1 is a mitochondrial division/mitophagy inhibitor. Treatment with Mdivi-1 suppressed PINK1 and Parkin expression [29,30]. In our study, Mdivi-1 down-regulated PINK1 in a dose-dependent manner and simultaneously promoted the replication of CVB3 (Figure 2(K, L)).

### Result 3. CVB3 non-structural protein 3C downregulates PINK1 expression and inhibits mitophagy

**Figure 3.**
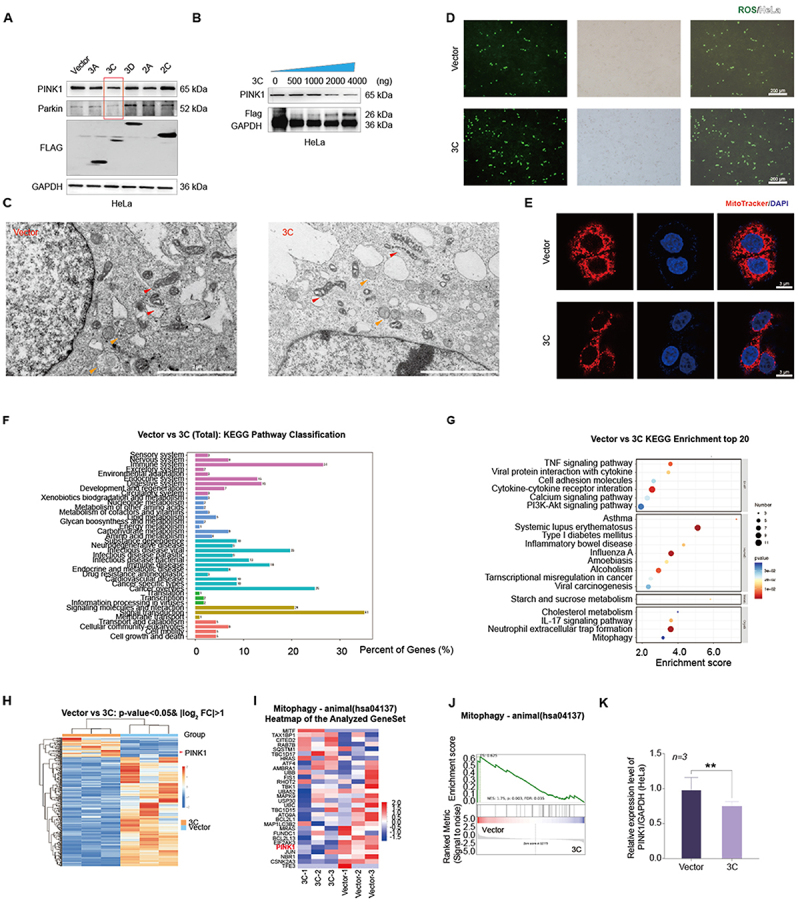
Down-regulation of PINK1 mediated by non-structural proteins 3C.

To identify viral effectors of PINK1 suppression, transfection assays in HEK-293T cells revealed that 3C, but not other non-structural proteins (2A, 2C, 3A, 3D), significantly reduced PINK1 and Parkin levels (Figure 3(A)). Dose-dependent studies in HeLa cells confirmed progressive PINK1 suppression with increasing 3C (Figure 3(B)). Notably, 3C-expressing cells recapitulated CVB3-induced mitochondrial defects (shrinkage, cristae disorganization; Figure 3(C)), alongside elevated ROS (Figure 3(D)) and mitochondrial fragmentation (Figure 3(E)).

A Western blot analysis showing the effects of non-structural proteins on PINK1, Parkin, and FLAG protein expression. B Dose-dependent effects of 3C protein on PINK1 protein expression levels by Western blot. C Representative TEM images of 3C-transfected HeLa cells, with pcDNA vector-transfected cells as negative control. Red arrows indicate mitochondria, yellow arrows indicate lysosomes. Scale bars = 2 μm. D Fluorescence microscopy analysis of ROS production in 3C-transfected HeLa cells. E MitoTracker staining demonstrating 3C-induced mitochondrial fragmentation in HeLa cells, with pcDNA vector-transfected cells as negative control. Scale bars = 3 μm. F and G KEGG pathway classification (F) and enrichment analysis (G) of differentially expressed genes in 3C-transfected HeLa cells. H Heatmap of differentially expressed genes in 3C-transfected HeLa cells. I Heatmap of single-sample gene set enrichment analysis (GSEA) scores for mitophagy-related genes in 3C-transfected cells. J GSEA analysis showing significant inhibition of the mitophagy pathway in 3C-transfected cells. K qRT-PCR analysis of PINK1 mRNA expression level in 3C-transfected HeLa cells. Data are presented as mean ± SD (n = 3 biological replicates per group). Statistical significance was determined by Student’s t-test (**P < 0.01).

RNA-seq of 3C-expressing HeLa cells highlighted enrichment in immune pathways, viral-host interactions, and mitophagy (Figure 3(F,G)). Transcriptome analysis heatmaps indicated reduced PINK1 expression in 3C-expressing cells (Figure 3(H)), and differential gene expression analysis also confirmed changes in mitophagy-related genes (Figure 3(I)). GSEA results supported these findings, showing the mitophagy pathway with an FDR =0.035 and a *P*-value=0.003 (Figure 3(J)). To validate 3C’s impact on PINK1 expression at the transcriptional level, qRT-PCR was performed, revealing a decrease in PINK1 mRNA expression following 3C expression (Figure 3(K)). Notably, neither 3C nor CVB3 cleaved PINK1 directly (Supplementary Figure S2A, S2B), and PINK1 mRNA declined progressively during infection (Supplementary Figure S2C).

### Result 4. 3C down-regulated PINK1 expression by FOSL1 binding to the PINK1 promoter region

**Figure 4.**
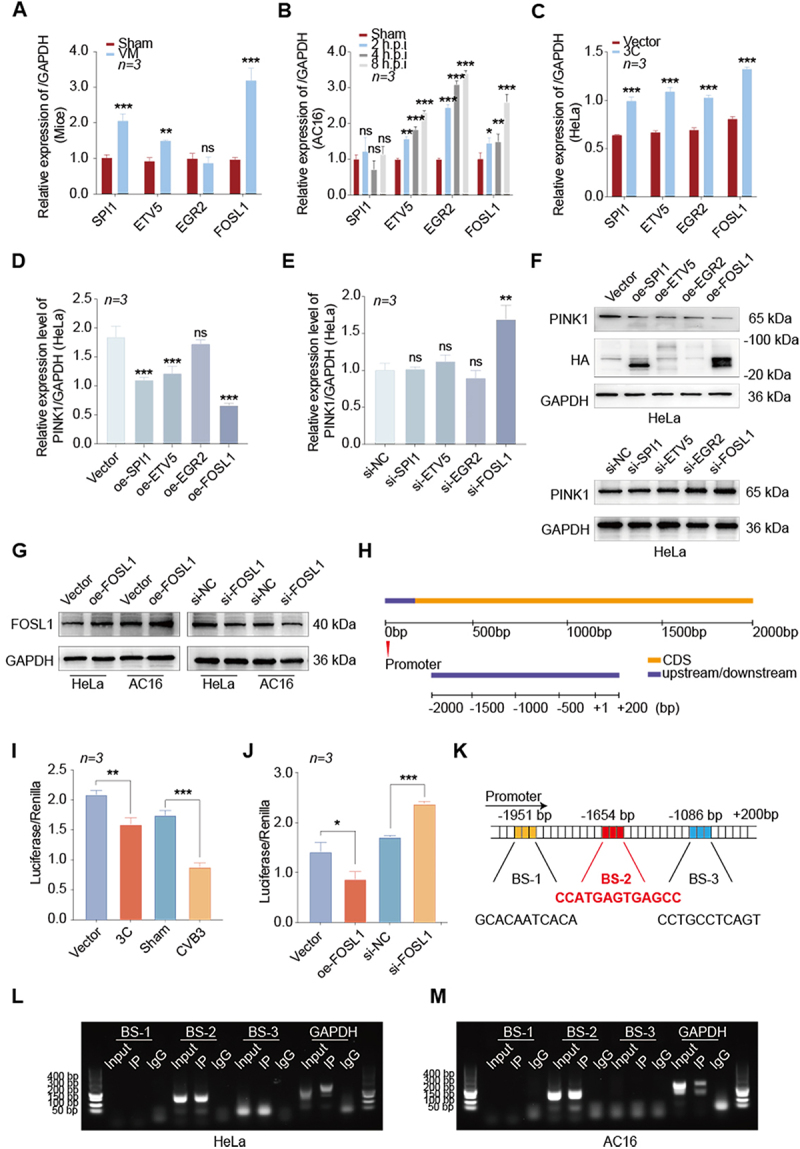
FOSL1-mediated down-regulation of PINK1 by 3C in CVB3 infection.

RNA-seq identified four 3C-regulated transcription factors (SPI1, ETV5, EGR2, FOSL1), all of which were up-regulated during CVB3 infection *in vivo* (Figure 4(A)). Our analysis of heart tissue from VM mice revealed that, except for EGR2, the other three transcription factors (SPI1, ETV5, and FOSL1) were up-regulated *in vitro* (Figure 4(B)). To verify whether these four transcription factors are consistent with the scRNA-seq results, we examined their expression in cardiomyocyte subsets. The results showed that, except for EGR2, SPI1, ETV5, and FOSL1 were differentially expressed in the VM group (Supplementary Figure S3A). Similarly, in HeLa cells, SPI1, ETV5, EGR2, and FOSL1 were all up-regulated after CVB3 infection (Supplementary Figure S3B). We further verified in cells that SPI1, ETV5, EGR2, and FOSL1 were all up-regulated following 3C transfection (Figure 4(C), HeLa cells; Supplementary Figure S3C, AC16 cells).

A qRT-PCR analysis the transcription factor expression levels in CVB3 infected VM mics group. B qRT-PCR analysis of transcription factor expression levels in AC16 cells. C qRT-PCR analysis of transcription factor expression level in HeLa cells transfected with 3C expression vector. D and E qRT-PCR analysis of PINK1mRNA expression in HeLa cells following overexpression (D) or silencing (E) of candidate transcription factors. F Western blot analysis of PINK1 expression in HeLa cells following overexpression or silencing of candidate transcription factors. G Western blot analysis of FOSL1 protein levels in HeLa and AC16 cells. H Schematic diagram of the PINK1 promoter region. I Luciferase reporter assay showing effects of 3C protease and CVB3 infection on PINK1 promoter activity. J Luciferase Reporter Assay showing reduced PINK1 promoter activity upon FOSL1 silencing. K Three potential FOSL1 binding sites (BS) within the PINK1 promoter region (JASPAR database: http://jaspar.genereg.net/). L and M ChIP-PCR agarose gel electrophoresis verified FOSL1 binding to PINK1 promoter in HeLa (L) and AC16 (M) cells. Red arrows indicate specific binding at BS2 (~150 bp bands). Input: total chromatin; IgG: negative control; GAPDH: positive control. Data were analyzed by two-way ANOVA and Student’s t-test. All experiments used biological replicates (n = 3/group). Data are represented as mean ± SD. *P < 0.05 **P < 0.01, ***P < 0.001.

To further investigate the impact of transcription factors on PINK1 mRNA expression, cells overexpressing or silencing transcription factors were constructed, respectively. qRT-PCR data indicated that overexpression of ETV5, EGR2, and FOSL1 reduced PINK1 mRNA expression (Figure 4(D)). However, only knockdown of FOSL1 could increase PINK1 mRNA expression levels (Figure 4(E)). Similarly, at the protein level, overexpression of SPI1, ETV5, EGR2, and FOSL1 all suppressed PINK1 expression, while only the knockdown of FOSL1 had a significant effect on increasing PINK1 expression (Figure 4(F)). These findings indicate that FOSL1 likely serves as a key transcription factor responsible for downregulating PINK1 expression during both CVB3 infection and 3C transfection.

Western blot and qRT-PCR analysis confirmed successful FOSL1 knockdown/overexpression in HeLa and AC16 cells via siRNA/plasmid transfection, respectively (Figure 4(G), Supplementary Figure S3D-S3E). Additionally, to verify whether FOSL1 affects CVB3 replication, we co-transfected with si-FOSL1 and the 3C vector in HeLa cells and infected them with CVB3. We found that si-FOSL1 significantly reduced CVB3 replication. However, this inhibitory effect was reversed when cells were co-transfected with si-FOSL1 and the 3C vector (Supplementary Figure S3F).

To validate this hypothesis, we first identified the PINK1 promoter region (−2000 to + 200 bp relative to the transcription start site, TSS) using the UCSC Genome database (Figure 4(H)). Potential interactions between FOSL1 and the PINK1 promoter were then predicted using the JASPAR database (http://jaspar.genereg.net/). Dual-luciferase reporter assays revealed that PINK1 promoter activity was significantly reduced in the 3C-transfected group compared to the Vector control, with further suppression observed upon CVB3 infection (Figure 4(I)). Consistent with its proposed role, FOSL1 overexpression decreased PINK1 promoter activity, while FOSL1 silencing enhanced it (Figure 4(J)). To pinpoint the precise binding site (BS) of FOSL1 within the PINK1 promoter, we performed chromatin immunoprecipitation (ChIP) assays targeting three predicted GC box motifs (BS1, BS2, and BS3; Figure 4(K)). ChIP-PCR analysis demonstrated that FOSL1 specifically binds to the BS2 region (CCATGAGTGAGCC, located at −1654 bp) but not to BS1 or BS3 in both HeLa (Figure 4(L)) and AC16 (Figure 4(M)) cells. These results indicate that FOSL1 directly represses PINK1 transcription by binding to a specific locus within its promoter region.

### Result 5. PINK1 suppresses CVB3 replication in vitro

**Figure 5.**
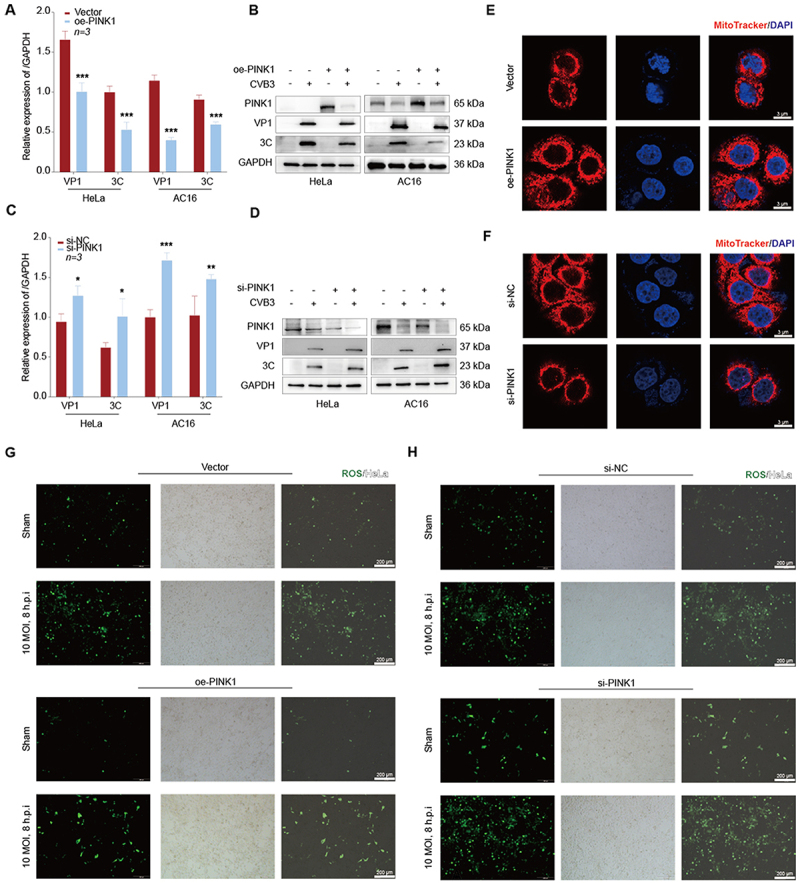
PINK1 suppresses CVB3 replication.

To investigate the role of PINK1 in CVB3 replication, HeLa and AC16 cells were transfected with an oe-PINK1 vector prior to CVB3 infection (10 MOI, 8 h.p.i for HeLa cells, and 50 MOI, 24 h.p.i for AC16 cells). Western blot and qRT-PCR to detect the overexpression and knockdown efficiency of PINK1 (Supplementary Figures S4A-S4D). qRT-PCR (Figure 5(A)) and Western blot (Figure 5(B)) were used to analyses of total RNA and protein revealed that PINK1 overexpression significantly suppressed VP1 and 3C expression. Conversely, PINK1 knockdown enhanced VP1 and 3C expression (Figure 5(C,D)). To further visualize PINK1 could suppress CVB3 replication, HEK-293T cells were transfected with oe-PINK1 and infected with GFP-CVB3. Fluorescence microscopy demonstrated a marked reduction in GFP-positive cells upon PINK1 overexpression (Supplementary Figures S4E, S4F). These results indicate that PINK1 suppresses CVB3 replication *in vitro*.

A and B qRT-PCR (A) and Western blot (B) analysis of VP1 and 3C expression in HeLa and AC16 cells following PINK1 overexpression. C and D qRT-PCR (C) and Western blot (D) analysis of VP1 and 3C expression in HeLa and AC16 cells following PINK1 silencing. E and F Mitochondrial morphology visualized by MitoTracker staining in HeLa cells following overexpression (E) or silencing (F) of PINK1, Scale bar = 3 μm. G and H Representative fluorescence microscopy images showing ROS production in HeLa cells after PINK1 overexpression (G) or silenced (H) followed by CVB3 infection (Scale bar = 200 μm). Data were analyzed by two-way ANOVA, n = 3/group. All data are represented as mean ± SD. *P < 0.05 **P < 0.01, ***P < 0.001.

Given the link between mitochondrial damage and ROS, we assessed the impact of PINK1 on mitochondrial function. PINK1 overexpression preserved mitochondrial integrity (Figure 5(E)) and reduced ROS (Figure 5(G)). Whereas PINK1 silencing induced mitochondrial fragmentation (Figure 5(F)) and elevated ROS levels (Figure 5(H)). Additionally, qPCR of mtDNA, a biomarker of mitochondrial damage, showed that PINK1 knockdown enhanced mtDNA expression, while PINK1 overexpression or 3C transfection had opposing effects (Supplementary Figures S4G-S4H). These findings implicate PINK1 in maintaining mitochondrial homeostasis and modulating antiviral responses.

### Result 6 MAVS mediates the antiviral effect of PINK1

**Figure 6.**
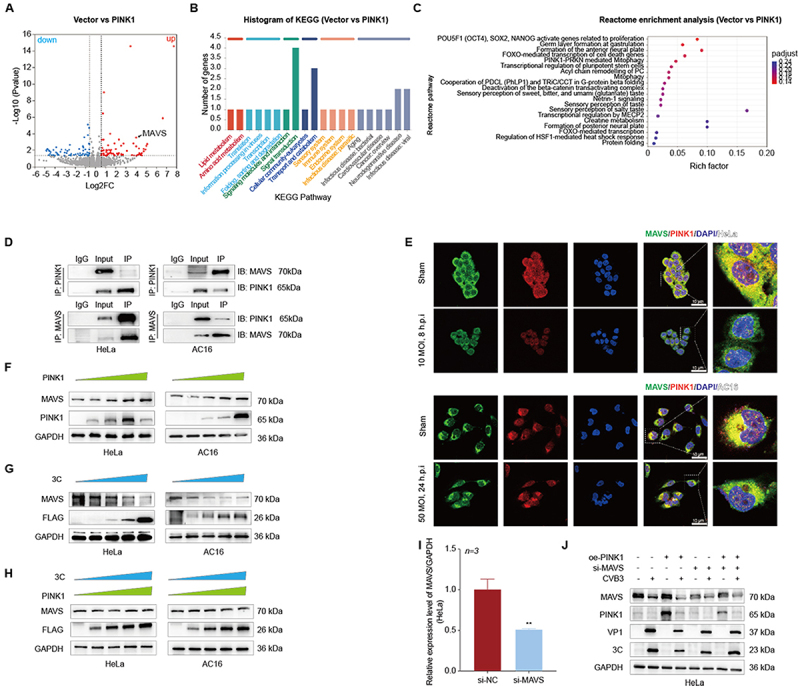
PINK1 regulates the antiviral immune response through MAVS.

To elucidate the underlying mechanism, we performed proteomic analysis of PINK1-overexpressing HeLa cells (vs. pcDNA control). Volcano plot (Figure 6(A)) analyses identified 143 differentially expressed proteins (Log2 Fold Change >2, *P* < 0.05, 73 upregulated, 70 downregulated). Notably, MAVS, a mitochondrial antiviral protein, was detected in PINK1-enriched immune complexes and upregulated upon PINK1 overexpression. KEGG pathway analysis linked these proteins to signal transduction, neurodegenerative diseases, and infectious diseases (Figure 6(B)), while Reactome enrichment highlighted roles in cell proliferation, mitophagy, apoptosis, and transcription (Figure 6(C)).

A Volcano plot illustrating differentially expressed genes in PINK1-overexpressing HeLa cells. B and C KEGG pathway classification (B) and enrichment (C) of differentially expressed proteins in PINK1-overexpressing HeLa cells. D Co-IP analysis of PINK1-MAVS interaction in HeLa and AC16 cells. E Representative immunofluorescence images showing MAVS (green) and PINK1 (red) co-localization in HeLa and AC16 cells, Scale bar = 10 μm. F Western blot analysis of PINK1 and MAVS expression in HeLa and AC16 cells following transfection with different concentrations of oe-PINK1 vector. G Western blot analysis of MAVS and FLAG expression in HeLa and AC16 cells following transfection with different concentrations of 3C-FLAG vector. H MAVS expression levels in HeLa and AC16 cells co-transfected with 3C and PINK1 expression vectors at indicated concentrations, as determined by Western blot. I qRT-PCR analysis the expression level of MAVS upon HeLa cells transfected with si-MAVS, used si-NC for negative control. J Western blot detection of MAVS, PINK1, VP1 and 3C expression upon HeLa cells co-transfected with si-MAVS and oe-PINK1 vector. Data were analyzed by t-test, n = 3/group. All data are presented as mean ± SD.**P < 0.01.

Co-IP assays in HeLa and AC16 cells confirmed PINK1-MAVS interaction (Figure 6(D)), and confocal microscopy showed their cytoplasmic colocalization and concurrent downregulation in HeLa and AC16 cells post-CVB3 infection (Figure 6(E)). Western blot analysis revealed that PINK1 overexpression dose-dependently enhanced MAVS levels in HeLa and AC16 cells (Figure 6(F)). In previous studies, protease 3C has been shown to downregulate the protein expression of MAVS by cleaving it [31]. In the present study, we also observed that 3C downregulates MAVS expression in a concentration-dependent manner (Figure 6(G)). Strikingly, co-expression of PINK1 reversed 3C-induced MAVS down-regulation (Figure 6(H)), suggesting a regulatory interplay between 3C and PINK1.

During viral infection, MAVS functions as a key mediator in activating the kinase complexes TBK1/IKK and TAK1-IKK. This activation is followed by phosphorylation of the transcription factors IRF3 and NF-κB, ultimately leading to the induction of type I interferons (IFNs) and downstream antiviral genes [32]. We co-transfected HeLa cells with si-MAVS (to knockdown MAVS) and the oe-PINK1 vector, then infected the cells with CVB3. Western blot analysis was performed to assess the effect of this treatment on viral replication. The results showed that MAVS knockdown reversed the inhibitory effect of PINK1 on viral replication, and these findings demonstrate that the inhibitory effect of PINK1 on viral replication is mediated by MAVS (Figure 6(I-J)).

## Discussion

**Figure 7.**
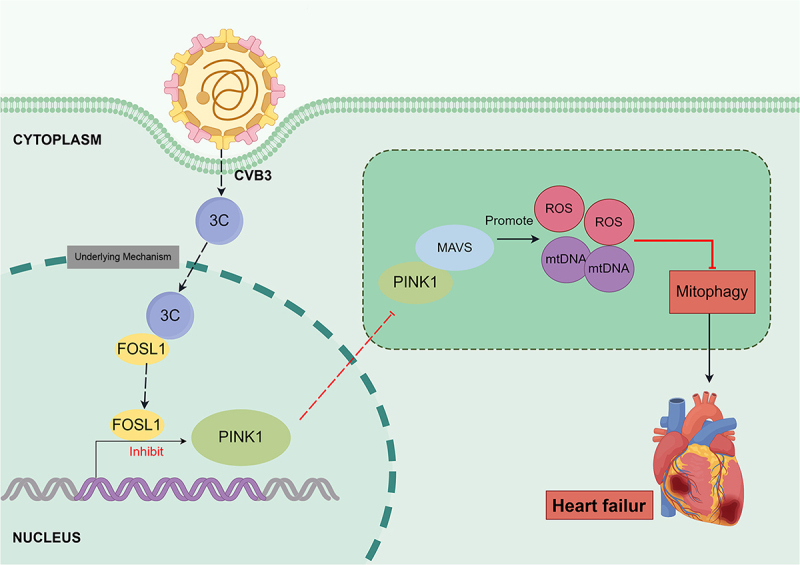
Mechanism schematic diagram.

Our investigation elucidates a novel molecular pathway through which CVB3 subverts mitochondrial antiviral defenses to promote viral myocarditis pathogenesis. Specifically, CVB3 employs its non-structural protein 3C to down-regulate PINK1 via the transcription factor FOSL1. This regulatory cascade results in the inhibition of PINK1-mediated mitophagy, which in turn facilitates viral replication. Furthermore, PINK1 exerts its antiviral effect against CVB3 by acting through MAVS. Downregulation of PINK1 leads to reduced MAVS protein expression; consequently, PINK1 loses its ability to inhibit CVB3 replication. Meanwhile, the suppression of PINK1 triggers an up-regulation of ROS and mtDNA, exacerbating mitochondrial dysfunction and amplifying cellular damage. In conclusion, CVB3 negatively regulates mitophagy via the 3C-FOSL1-PINK1-MAVS axis, thereby impairing mitochondrial function and facilitating viral replication (Figure 7). These findings substantially advance our understanding of CVB3-host mitochondrial interactions and identify critical nodes for therapeutic intervention in VM.

Proposed mechanism of the 3C/FOSL1/PINK1/MAVS axis in CVB3-induced VM: 3C downregulates PINK1 expression through FOSL1, while simultaneously inhibiting MAVS expression through both direct action and PINK1 suppression.

Enteroviruses have evolved to hijack autophagy, a catabolic recycling process that degrades protein aggregates, damaged organelles, and invades microbes such as viruses. Selective autophagy relies on autophagy receptors including optineurin (OPTN), Tax1-binding protein 1 (T6BP), and SQSTM1, which bridge microtubule-associated protein 1 light chain 3 (LC3) to both ubiquitinated and non-ubiquitinated substrates [33]. Autophagosome formation involves two ubiquitin-like conjugation systems with maturation occurring through fusion with lysosomes for cargo degradation, a process monitored by LC3B lipidation and SQSTM1 turnover. Enterovirus D68 exploits this pathway by blocking autophagosome-lysosome fusion to enhance its replication [34]. Similarly, previous studies have shown that CVB3 impedes lysosomal degradation through fusion blockade, thereby enhancing viral release [35,36]. Nevertheless, the precise mechanisms through which CVB3 stimulates autophagosome formation while inhibiting its degradation during viral myocarditis remain unclear.

Increased autophagosome formation has been observed in CVB3-infected HeLa cells [36]. Regarding autophagy receptors and autophagosome biogenesis, CVB3 non-structural protein 2B can directly induce autophagosome formation [37], whereas the viral 2A protease cleaves SQSTM1 to reduce its abundance facilitating viral replication and release [38]. Notably, the interaction between CVB3 and autophagy follows distinct temporal dynamics. Early in infection, CVB3 particles are enveloped by autophagosomes and delivered to lysosomes, activating a positive-feedback loop of autophagy. As infection progresses to the late stage, CVB3 disrupts lysosomal function, impairing autophagosome degradation and leading to SQSTM1 accumulation [39]. This stage-specific regulation suggests that selective autophagy operates in different modes during CVB3 pathogenesis, making the overall role of autophagy in disease progression highly complex.

3C, a key protease of CVB3, has been well-documented in previous studies to regulate the expression of host proteins through cleavage, thereby influencing viral replication and the host’s antiviral immune response. However, the present study identifies a novel mechanism: 3C downregulates PINK1 expression at the transcriptional level by upregulating the transcription factor FOSL1—a regulatory mode that differs from its typical cleavage-dependent modulation of host proteins and has been rarely reported. Notably, how 3C specifically upregulates FOSL1 remains an open question worthy of further discussion. Previous studies have shown that YTHDF2 can negatively regulate the expression of FOSL1. From a mechanistic perspective, YTHDF2 promotes FOSL1 mRNA decay in an m6A-dependent manner, which in turn upregulates FOSL1 expression [40]. Additionally, other studies have indicated that YTHDF2 can be downregulated via cleavage by 2A [41]. As a protease also with cleavage activity, does 3C exert the same effect as 2A? To address this question, we transfected 3C vector into HeLa cells. Western blot analysis of YTHDF2 revealed that 3C could downregulate its expression, but no cleavage band was observed (data not shown). This suggests that both 3C and 2A can downregulate YTHDF2, yet the specific mode of action of 3C remains unclear. Based on the above experimental results and literature, we can hypothesize that 3C upregulates FOSL1 expression in an m6A-dependent manner by downregulating YTHDF2 expression; however, the specific mechanism of action still requires further investigation.

Although PINK1 and Parkin are primarily recognized for their roles in mitophagy, emerging evidence indicates their involvement in mitochondrial quality control is also linked to innate immunity [42]. MAVS, a mitochondrial membrane protein, is essential for activating the IFN response [43]. Numerous viruses have evolved strategies to disrupt MAVS signaling and interfere with RLR signaling to evade innate immune defenses [13,44]. In our study, knockdown of MAVS reverses the inhibitory effect of PINK1 on CVB3 replication, which indicates that MAVS is critical for the antiviral function of mitophagy. In addition, the 3C-induced mtDNA efflux observed in our study establishes a critical link between viral mitochondrial manipulation and cardiac remodeling. Through PINK1 suppression, 3C exacerbates mtDNA-driven activation of cGAS-STING signaling – a known accelerator of cardiac hypertrophy and inflammation [45]. In our study, the presence of 3C alone was sufficient to induce mtDNA release. By suppressing PINK1 expression, 3C further amplified mtDNA release, contributing to the progression of infected cardiomyocytes toward DCM. This mechanism provides a plausible explanation for the progression from VM to DCM, particularly in the context of chronic mtDNA release and sustained innate immune activation.

Heart failure, a complex clinical syndrome, is primarily caused by cardiac fibrosis, VM, as well as other pathological conditions such as DCM [46]. Therefore, therapeutic intervention for VM is crucial for reducing the incidence and progression of HF. Notably, our study reveals that PINK1 exerts its antiviral effect not only by activating mitophagy to inhibit viral replication but also by facilitating MAVS-related pathways. Mitophagy is able to minimize the functional decline of aging cardiomyocytes by degrading and recycling long-lived proteins, as well as cytoplasmic components and dysfunctional organelles. Mitophagy might also be able to counteract cardiac aging, and maintain myocardial function. Urolithin A, NAD+ precursors, and spermidine as activators stimulates mitophagy play an important role in cardiomyopathy [47–51]. Similarly, PINK1 serves as a key initiator protein of mitophagy, playing a crucial role in activating this process; it may thus hold significant clinical value for the future treatment of cardiomyopathy.

Our study utilized HeLa, AC16 and HEK-293T cells, although these models clarified the axis’s regulatory role, they have limitations: non-cardiac cells lack myocardial-specific traits to reflect mitophagy-cardiac function crosstalk. AC16 cells show impaired mitochondrial stress responses. In addition, murine models differ from humans in CVB3 tropism and immune profiles, restricting clinical translation [52]. Induced pluripotent stem cell (iPSC)-derived cardiomyocytes better recapitulate human myocardium – they retain donor genetics, differentiate into mature cells with physiological functions, and successfully model human CVB3-induced VM [53]. Future studies will use these cells to validate the 3C-FOSL1-PINK1-MAVS axis in human contexts and test interventions, enhancing clinical relevance.

In conclusion, this study elucidates a complex interplay between CVB3, its non-structural protein 3C, and the host mitochondrial antiviral response. Specifically, CVB3 utilizes 3C to transcriptionally suppress PINK1 via upregulating the transcription factor FOSL1; reduced PINK1 expression subsequently downregulates the mitochondrial antiviral protein MAVS and impairs mitophagy. This dual disruption of the PINK1-MAVS axis not only compromises the host’s antiviral defense but also promotes mtDNA release, a key trigger for increased cardiac inflammation, ultimately driving the progression of VM toward DCM. These findings provide novel insights into the mechanisms by which CVB3 actively manipulates host cellular processes to facilitate its own replication and exacerbate cardiac pathogenesis. More importantly, they highlight the PINK1-MAVS axis as a potential therapeutic target for mitigating CVB3-induced cardiac damage and preventing the transition from VM to heart failure.

## Supplementary Material

ARRIVE guidelines 2 English20250701.pdf

CleanSupplementary_information_20260211.docx

## Data Availability

The scRNA-sequencing data of viral myocarditis have been deposited in the Genome Sequence Archive (GSA, https://ngdc.cncb.ac.cn/gsa/, CRA035188) and are publicly accessible without restrictions. The RNA-seq data of 3C-Vector transfected cells have been uploaded to the Genome Sequence Archive for Human (GSA-Human, https://ngdc.cncb.ac.cn/gsa-human/, HRA015193) with open access to all qualified researchers in accordance with human genetic resource management regulations. All genome-related datasets, along with other data supporting the findings of this study, including raw data from mouse infection experiments, additional RNA-seq data, and source data for figures and tables, are openly available on ScienceDB under a CC-BY 4.0 International license. The datasets generated during and/or analyzed during the current study are available in the ScienceDB (https://doi.org/10.57760/sciencedb.31524) repository, reference number [54]. No data in this study are subject to embargo; all datasets are either already publicly accessible or will be released immediately upon manuscript acceptance, consistent with institutional and data repository policies for open scientific data sharing.
