# Supplementary material for: 3C suppresses PINK1-mediated mitophagy and contributes to coxsackievirus B3 replication
Source: Virulence. 2026 Apr 26;17(1):2662767. doi: 10.1080/21505594.2026.2662767 (PMC13114115; doi:10.1080/21505594.2026.2662767)
Supplement: CleanSupplementary_information_20260211.docx [file KVIR_A_2662767_SM6899.docx]

**Supplementary information**

**3C suppresses PINK1-mediated mitophagy and contributes to Coxsackievirus B3 replication**

Authors: Tingjun Liu^1†^, Ao Wan^2,3†^, Yinhai Xu^1†^, Hongxiang Lv^5^, Yiwei Xie^1,2^, Han Wu^2^, Jiang Wang^2^, Hua Wang^4^, Tingting Hao^1^, Yonggen Zhang^1^, Jinfeng Xu^6^, Hongxing Shen^4*^, Shibao Li^1,2*^

^1^ Department of Laboratory Medicine, The Affiliated Hospital of Xuzhou Medical University, Xuzhou, 221006, China.

^2^ School of Medical Technology, Xuzhou Medical University, Xuzhou, 221002, China.

^3^ Department of Laboratory Medicine, The people’s Hospital of Jiawang, Xuzhou, 221011, China

^4^ Department of Laboratory Medicine, School of Medicine, Jiangsu University, Zhenjiang, 212013, China

^5^ Department of Laboratory Medicine, Jiangning Hospital Affiliated to Nanjng Medical University, Nanjing 211100, China

^6^ Department of Clinical laboratory, Zhenjiang center for disease control and prevention, Zhenjiang, Jiangsu, 212000, China.

^†^ Tingjun Liu, Ao Wan, and Yinhai Xu have contributed equally to this work and share the first authorship.

* Corresponding author: ^1^Shibao Li, E-mail: sdjnshlb@xzhmu.edu.cn, School of Medical Technology, Xuzhou Medical University, Xuzhou, 221002, China.

^2^Hongxing Shen, E-mail: hxshen@ujs.edu.cn, Department of Laboratory Medicine, School of Medicine, Jiangsu University, Zhenjiang, 212013, China

**Supplementary information content:**

**1. Supplementary Figures with figure legends**

**2. Supplementary Tables**

**1. Supplementary Figures with figure legends**

**Supplementary Figure 1. KEGG pathway enrichment analysis of cluster-specific genes in cardiomyocyte subpopulations.**

**A** KEGG pathway analysis of differentially expressed genes specific to cluster 1 cardiomyocytes. **B** KEGG pathway analysis of differentially expressed genes specific to cluster 2 cardiomyocytes. **C** KEGG pathway analysis of differentially expressed genes specific to cluster 4 cardiomyocytes.

**Supplementary Figure 2. PINK1 expression analysis in HeLa cells under different conditions.**

**A** Western blot analysis of PINK1 protein expression in 3C protease-transfected HeLa cells. **B** Western blot analysis of PINK1 protein expression in CVB3-infected HeLa cells (MOI=10, 8 h post-infection). **C** qRT-PCR analysis of PINK1 mRNA levels in CVB3-infected HeLa cells (MOI=10, 8 h post-infection). Data analysed by two-way ANOVA, the n values are all biological replicates, n = 3/group. All data expressed as mean ±SD. **P* < 0.05, ***P* < 0.01.

**Supplementary Figure 3. FOSL1 expression analysis and its function on CVB3 replication.**

**A** ScRNA-seq was used to analyze the expression levels of the transcription factors SPI1, ETV5, EGR2, and FOSL1. **B** qRT-PCR analysis the transcription factor expression levels in CVB3 infected HeLa cells. **C** qRT-PCR analysis the transcription factor expression level in AC16 cells transfected with 3C expression vector. **D and E**. qRT-PCR to detected the expression level of FOSL1 upon HeLa cells transfected oe-FOSL1 (**D**) or si-FOSL1 vector (**E**), used Vector or si-NC for negative control. **F** Western blot to analysis the function of si-FOSL1 on CVB3 replication. Data analysed by two-way ANOVA, the n values are all biological replicates, n = 3/group. All data expressed as mean ±SD. **P* < 0.05, ***P* < 0.01, ****P* < 0.001.


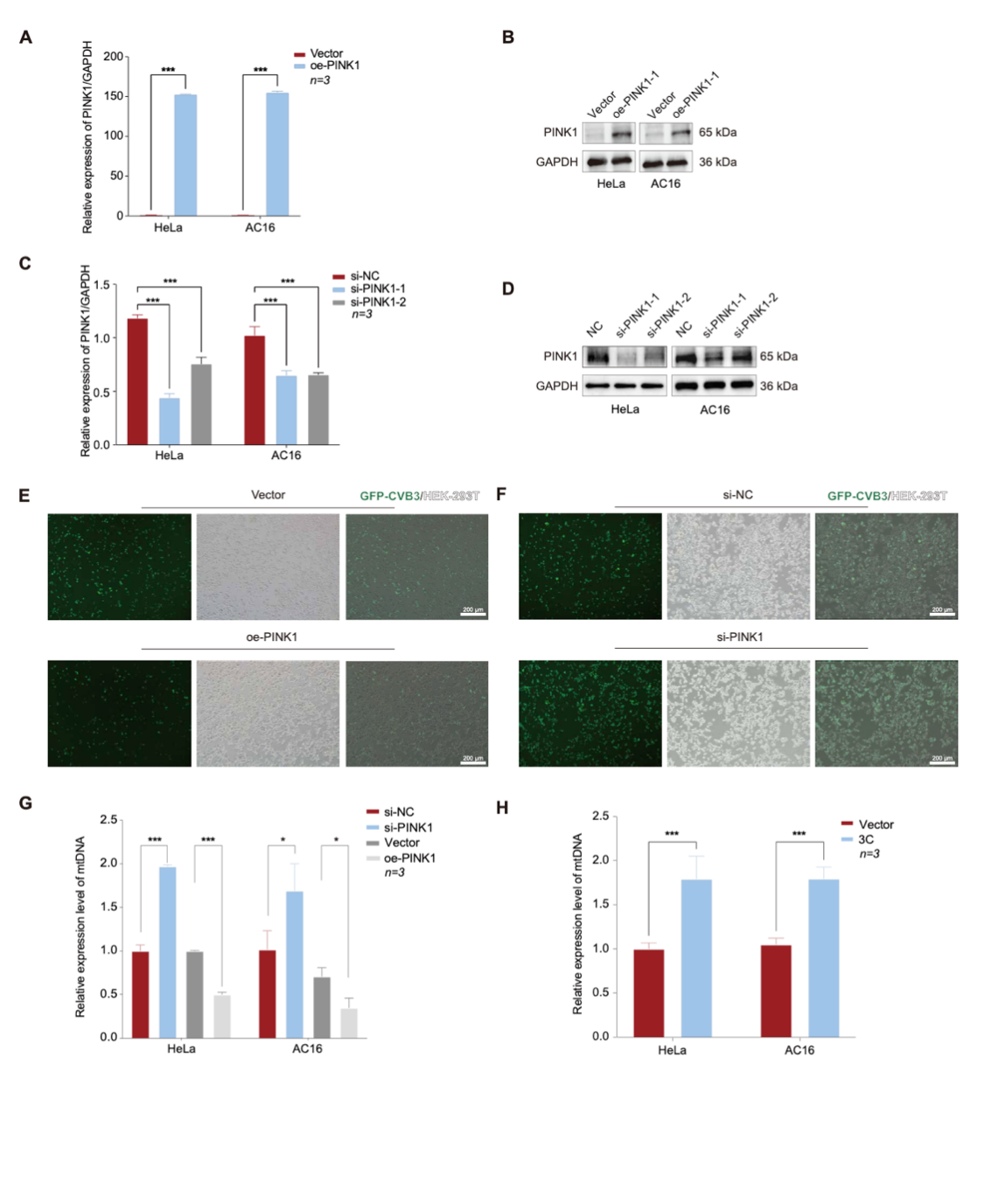


**Supplementary Figure 4. Validation of PINK1 function on GFP-CVB3 replication and mtDNA released.**

**A** qRT-PCR analysis of PINK1 overexpression efficiency. **B** Western blot analysis of PINK1 protein levels after overexpression in cells. **C** qRT-PCR analysis of PINK1 silenced efficiency. **D** Western blot analysis of PINK1 protein levels after silenced in cells. **E** Fluorescence microscopy images showing GFP expression in HEK-293T cells overexpressing PINK1. **F** Fluorescence microscopy images showing GFP expression in HEK-293T cells with PINK1 knockdown. **G** Relative mitochondrial DNA (mtDNA) copy number in HeLa and AC16 cells following PINK1 overexpression or silencing. **H** Relative mtDNA copy number in HeLa and AC16 cells expressing 3C protease. In both panels, 18S rRNA served as the loading control. Data were analyzed by two-way ANOVA with biological replicates (n = 3 per group) and are presented as mean ± SD. * *P* < 0.05 , ** *P* < 0.01, ****P* < 0.001

**3. Supplementary Tables**

Supplementary Table 1. The primers sequences of mtDNA

| Primer | Sequence (5’-3’) |
| --- | --- |
| mtDNA | CACCCAAGAACAGGGTTTGT |
|  | TGGCCATGGGTATGTTGTTAA |
| 18S | TAGAGGGACAAGTGGCGTTC |
|  | CGCTGAGCCAGTCAGTGT |
